# Long Non-Coding RNAs as “MYC Facilitators”

**DOI:** 10.3390/pathophysiology30030030

**Published:** 2023-09-01

**Authors:** Daniel García-Caballero, Jonathan R. Hart, Peter K. Vogt

**Affiliations:** Department of Molecular Medicine, The Scripps Research Institute, 10550 North Torrey Pines Road, La Jolla, CA 92037, USA

**Keywords:** lncRNAs, CRISPRi, CRISPR–Cas9, transcription factor–RNA complex, transcriptional regulation

## Abstract

In this article, we discuss a class of MYC-interacting lncRNAs (long non-coding RNAs) that share the following criteria: They are direct transcriptional targets of MYC. Their expression is coordinated with the expression of MYC. They are required for sustained MYC-driven cell proliferation, and they are not essential for cell survival. We refer to these lncRNAs as “MYC facilitators” and discuss two representative members of this class of lncRNAs, SNHG17 (small nuclear RNA host gene) and LNROP (long non-coding regulator of POU2F2). We also present a general hypothesis on the role of lncRNAs in MYC-mediated transcriptional regulation.

## 1. Introduction

MYC proteins are a family of transcriptional regulators that, in the human genome, are encoded by three genes: c-MYC, N-MYC, and L-MYC [1]. We will refer to these collectively as MYC. MYC is a representative of a larger group of proteins that share characteristic structural elements consisting of a basic domain, a helix–loop–helix domain, and a leucine zipper, which are referred to as bHLH LZ proteins [2]. The interaction of MYC with DNA requires dimerization with one of several related small bHLH LZ proteins, generally with MAX [3,4,5], and is targeted to a DNA sequence referred to as the E-box [6].

The predominant regulatory function of MYC consists of the universal amplification of all active transcription [7,8,9,10]. Therefore, MYC is not a transcription factor, as it is commonly thought of, with a limited set of target genes. It regulates transcription on a broader scale, although the possibility of selective interactions with specific promoters has not been ruled out [7]. An example of this selectivity is MYC-induced oncogenic cellular transformation, which is characterized by a unique expression profile [11,12,13]. MYC can affect almost every cellular activity, including proliferation, differentiation, migration, metabolism, and apoptosis [14,15,16,17,18]. MYC is not confined to intracellular activity; it can also extend its influence to the extracellular space by interfering with tumor immunity [19,20]. The diversity and multiplicity of MYC-mediated interventions in cellular processes are probably facilitated by structural features of the MYC protein that include intrinsically disordered regions. Such regions can mediate interactions with an exceptionally large number of partner proteins [7,21].

MYC gain of function is broadly growth-stimulatory. This ability is reflected in an overt oncogenic potential, which was a key factor in the discovery of MYC as a retroviral oncogene [22]. Since then, MYC has emerged as one of the most important drivers of human cancers. In contrast to other proto-oncogenes whose carcinogenic functions invariably depend on mutation, MYC is either over-expressed or amplified in human cancer, but it does not acquire gain of function by mutation [23,24,25].

As a predominant cancer driver, MYC is an important drug target [26]. However, like other transcriptional regulators, MYC lacks defined pockets or grooves that can serve as high-energy interaction sites for small molecules. Standard approaches that are successful in the inhibition of enzymes cannot be applied to MYC. Several alternative strategies have been used, yielding effective inhibitors that currently serve as valuable experimental tools. Importantly, they have invalidated the widespread assumption that MYC is “undruggable”. Recent publications have provided a comprehensive assessment of the available data on MYC inhibitors and new directions [11,17,27,28,29,30]. However, the path to the clinic still seems daunting and long. Recognizing the obstacles that impede the success in direct MYC inhibition, indirect interference with MYC activity has been pursued by blocking the function of critical downstream mediators of MYC. This approach has yielded new means of controlling MYC activity [31,32,33]. Furthermore, indigenous cellular circuits that can attenuate MYC have been proposed and deserve additional study [34].

This review deals primarily with non-coding RNAs. Non-coding sequences are found in all metazoans; they represent a portion of the transcriptome that increases with the organizational complexity of an organism [35]. More than 98% of the human transcriptome consists of non-coding RNAs [2,36]. The field of non-coding RNAs and their significance has been an area of contention for decades. On the one hand, there is the view that non-coding sequences, including introns, are remnants of evolution, representing DNA that has no function except generating transcriptional noise [37]. On the other hand, there is growing evidence for critically important functions of the non-coding genome. This evidence emerged from deep sequencing and the functional analysis of individual transcripts. This debate has recently culminated in compelling papers that urge fundamental changes in our thinking on genes, development, and evolution [38,39], but criticism and doubts persist [37,40].

A large segment of the non-coding transcriptome is classified as lncRNAs (long non-coding). lncRNAs were originally and arbitrarily defined as encompassing transcripts of more than 200 nucleotides in length. A more recent definition has moved this minimal size up to 500 nucleotides [35]. The key properties of lncRNAs are as follows: (1) specificity for tissue, cell lineage, and cell type; (2) generally low levels of expression; (3) modular structure; and (4) a higher rate of evolution than protein-coding genes [39]. A large portion of lncRNAs share an important function with MYC: regulating transcriptional activity. They function as scaffolds and as recruiters directing chromatin-modifying enzymes to specific sites of the genome. They can also interact with transcription factors [39,41,42,43]. The connection between the expression of MYC and lncRNAs first came to light in two studies that used the human B cell line P493-6 [44]. In this cell line, MYC acts as an integrated external transgene that is under the control of a Tet-off promoter [45,46]. This cell line expresses high levels of MYC, facilitating rapid cell proliferation. In the presence of the doxycycline repressor, the level of MYC drops by a factor of more than 40, and cell proliferation becomes barely detectable [44]. In one of these early studies, about 1300 lnc transcripts were identified, of which 42% responded to the expression level of MYC [46]. This dependence on MYC was validated by consulting available ChIP-seq (chromatin-immunoprecipitation-sequencing) data for the transcriptional start sites of the regulated lncRNAs and by verifying the binding of MYC to these sites. The observations presented in this study are in broad agreement with the transcriptional amplifying function of MYC. They establish a link between MYC, lncRNA expression, and cell proliferation, raising the question of the role of the lncRNA–MYC network in cancer. This general question has been illuminated by several recent reviews [26,45,47,48,49,50,51,52].

The purpose of this paper is to draw attention to a specific subgroup of lncRNAs. The transcription of these lncRNAs is stimulated by MYC, and their expression is required for sustained MYC-driven cell proliferation. We will refer to these RNAs as “MYC facilitators”.

## 2. Main text

### 2.1. Technical Considerations

The characterization of lncRNAs invariably leads to questions of methods and techniques. The commonly used approaches employ various kinds of mutagenesis or aim for a down- or upregulation of lncRNA expression. The goal is to link a phenotype to a mutational event or a changed expression pattern. Establishing such a link remains the gold standard for an initial characterization of a lncRNA.

For both mutagenesis and altered expression, the difficulties lie in the details. Mutating lncRNAs usually follow some form of CRISPR–Cas system [53,54]. However, an agnostic application of CRISPR–Cas9 is bound to be unsuccessful, because mutational inactivation of a lncRNA requires knowledge of the functionally essential domains of the molecule. In the absence of such knowledge, it is necessary to scan the entire sequence of the RNA with multiple sgRNAs (single guide RNAs) to identify a combination that mediates the successful inactivation of the target lncRNA. CRISPRi is the standard tool for downregulating the expression of a lncRNA [55,56,57], but it comes with its own set of specific difficulties. The widely used KRAB domain that provides repressor activity to dCas9 (inactivated Cas9) shows very low activity in some cell types. Thus, it is necessary to search for an alternative repressor domain. A recent example is dCas9-SID, an MXD1-derived SID repressor construct for use in human B cells [58]. A more general problem that affects mutagenesis and altered expression levels derives from the fact that most lncRNAs have a regulatory function, and changes in such functions can have only a subtle effect on the cellular phenotype. The solution to this problem is to use a guide competition assay. For instance, to validate a top hit identified by CRISPRi using the dCas9-SID construct, selected guide sequences are cloned into GFP-expressing lentiviral guide vectors, marking cells that express these sgRNAs. Cells carrying dCas9-SID are transduced with single fluorescent sgRNAs targeting the gene of interest and are grown in competition with non-transduced cells that express the dCas9-SID repressor construct only. If the targeted lncRNA is needed for optimal cell proliferation, the fraction of fluorescent cells decreases over time. The process is standardized with control competition assays using fluorescent sgRNAs that target essential cellular genes [59]. The results from such competition assays must agree with the corresponding CRISPRi data.

New techniques applicable to the characterization of lncRNAs are continuously being developed. Recent advances have emphasized the genome-wide annotations of lncRNAs. Examples of such advances include GRID-seq, RADICL-1-seq, and RIC-seq [60,61,62,63]. Purely computational methods have also been applied, revealing insightful correlations [64,65,66].

### 2.2. SNHG17

As the first representative of MYC facilitators, we present SNHG17 (small nucleolar RNA host gene 17). SNHG17 is a lncRNA consisting of 1186 nucleotides. It belongs to the SNHG family of genes and is situated on human chromosome 20q11.23. Building on previous studies that had revealed the widespread and probably general regulation of lncRNA transcripts by MYC, we applied CRISPRi to identify lncRNAs that are essential for MYC-driven cell proliferation in the P493-6 and Ramos B cell lines [58]. Guides targeting SNHG17 were consistently depleted from the library in these screens and in competition assays, confirming the importance of SNHG17 for cell proliferation and survival. Furthermore, ChIP-qPCR experiments demonstrated that SNHG17 is a direct transcriptional target of MYC, with MYC binding to its promoter region. Concurrently, several studies were published that investigated the role of SNHG17 in cancer. This work revealed multiple functions of SNHG17, implicating it in several cancer hallmarks, including enhanced proliferation, the invasion of neighboring tissues, the stimulation of angiogenesis, the migration and formation of metastases, and interference with the apoptosis pathway [67,68,69,70,71,72,73,74]. SNHG17 was first reported by Ma et al. [75], who discovered that SNHG17 overexpression in colorectal cancer led to increased cell proliferation through the epigenetic silencing of P57. Subsequent research found that SNHG17 was upregulated in numerous tumor types, including gastric and lung cancer, osteosarcoma, melanoma, and hepatocellular carcinoma. High SNHG17 expression levels are generally associated with a poor prognosis [76].

SNHG17 comprises various different transcripts and is expressed in the nucleus and cytoplasm of different tumor cells. SNHG17 can act as ceRNA (competing endogenous RNA). As a ceRNA, SNHG17 interacts with specific miRNAs (microRNAs) to modulate their target genes and, subsequently, influence key signaling pathways involved in cancer progression. In OSCC (oral squamous cell carcinoma), SNHG17 acts as a competing endogenous RNA for miR-384, upregulating ELF1, a transcriptional activator of CTNNB1, which, in turn, activates the Wnt/β-catenin signaling pathway and promotes OSCC development [77]. In melanoma, SNHG17 is upregulated by the transcription factor STAT3, contributing to melanoma progression by enhancing PI3K-AKT signaling and its growth-promoting effects that also facilitate cellular invasiveness and migration [78]. In ovarian cancer, the transcription factor STAT3 directly binds to the promoter region of SNHG17 and activates its transcription. SNHG17 then advances cell cycle progression by serving as a ceRNA of miR-214-3p, which results in an upregulation of CDK6 [79]. In hepatocellular carcinoma, SNHG17 promotes cancer hallmarks and epithelial–mesenchymal transition by interfering with the control of RFX1 (regulatory factor X1) by serving as a ceRNA of miR-3180-3p, which, in turn, leads to the overexpression of RFX1, a growth-stimulatory transcription factor with oncogenic potential that controls the expression of several cancer-relevant proteins in a cell [80]. In gastric cancer, SNHG17 acts as an oncogene by epigenetically silencing the tumor suppressor genes p15 and p57 through the activation of EZH2, a histone methyltransferase. The inactivation of p15 and P57 leads to enhanced cell proliferation, invasion, and migration [73]. Additionally, SNHG17 has been reported to play unique biological roles in two diabetic conditions, i.e., diabetic nephropathy and gestational diabetes [81,82].

Some of this multiplicity of functions and activities can probably be ascribed to differences in the techniques and approaches used by different laboratories. But a more fundamental explanation for this unusual diversity of SNHG17 action could be that we are dealing with different SNHG17 entities that are generated by cell- and tissue-specific alternative splicing [83]. Alternatively spliced lncRNAs have different functions. The SNHG17 identified in P496-3 and Ramos cells and the SNHG17s studied in various specific cancers, nephropathies, and diabetes are probably not the same and are unique in structure and function. They are SNHG17s by name only.

### 2.3. LNROP

LNROP (long non-coding regulator of POU2F2) is a transcript that was identified in a CRISPRi screen of MYC-regulated lncRNAs essential for B cell proliferation [84]. In the genome, it is situated in an antisense orientation to POU2F2, which is the gene that encodes the transcription factor OCT2 (octamer-binding protein 2). As indicated by its gene name, POU2F2, OCT2 is a member of the POU transcription factor family; it regulates transcription by binding DNA through specific POU domains. OCT2 is an essential regulator of immunoglobulin gene promoters and plays a key role in the proliferation and differentiation of human B cells. Furthermore, OCT2 expression is critical for the proliferation and survival of diffuse large B cell lymphomas [85,86,87,88].

The relationship between the expression of LNROP and OCT2 was analyzed using CRISPRi in combination with competition growth assays. This study demonstrated that LNROP regulates OCT2, with LNROP downregulation leading to a concomitant decrease in OCT2 expression. These CRISPRi experiments raise a potential concern: The LNROP and OCT2 genes are located in close proximity with shared promoter regions oriented in a head-to-head configuration. LNROP-targeted CRISPRi could directly interfere with the promoter of OCT2, and the observed effects could be due to the off-target activity of the sgRNA constructs. To clarify this issue, CRISPR–Cas9 was employed to induce LNROP loss of function. Dealing with a lncRNA, the CRISPR–Cas9 approach had to be modified by scanning the entire length of the LNROP gene using a library of several hundred sgRNAs. Several guides capable of interfering with LNROP function were identified by growth competition assays. They reduced the expression of LNROP and OCT2 concomitantly, supporting the conclusion that LNROP is a direct regulator of OCT2 and that CRISPRi data do not reflect an off-target effect caused by the proximity of the LNROP and OCT2 genes.

LNROP is a nuclear RNA, and ChIP-seq data show that it is a direct target of MYC. The disruption of LNROP or OCT2 leads to transcriptomic alterations. These include several pathways controlled by OCT2, as shown by gene set enrichment analysis. One of the downregulated targets of the LNROP–OCT2 axis is SHP-1. SHP-1 is a protein-tyrosine phosphatase that negatively affects cell proliferation by modulating signaling pathways initiated by growth factors, cytokines, and ITAM-containing receptors [89,90,91,92,93,94,95,96]. The LNROP–OCT2-mediated downregulation of SHP-1 reduces this growth-inhibitory effect of SHP1 and is in accord with the essential role of LNROP in the MYC-driven proliferation of B cells. The CRISPR–Cas9-mediated disruption of LNROP also interferes with the function of OCT2, but the disruption of OCT2 does not affect the expression of LNROP. The relationship between LNROP and OCT2 is thus unidirectional.

## 3. Concluding Considerations

lncRNAs have emerged as crucial regulatory molecules [38,39,97,98,99]. In the last few years, an intricate network between MYC and lncRNAs has come into view; it presents many of the features of symbiotic relationships [48,52,100,101].

The association between lncRNAs and MYC is a significant factor in oncogenesis; some lncRNAs enhance and others inhibit MYC transcriptional activity. Specific cancers provide examples of these effects. In prostate cancer, lncRNA RP11-1023L17.1 acts as an oncogene, regulating MYC’s transcriptional signature, and its depletion leads to the downregulation of MYC-dependent oncogenic features in a tumor [102]. In non-small cell lung cancer, lncRNA HIF1A-As2 forms a positive feedback loop with MYC, driving cell proliferation and tumor metastasis [103]. Similarly, in hepatic progenitor cells, lncRNA SNHG12 promotes proliferation and migration via the Wnt/β-catenin pathway, thereby changing MYC expression [104]. In bladder cancer, lncRNA SNHG18 inhibits MYC expression by modulating the ubiquitination–proteasome pathway, resulting in the suppression of cancer cell growth [105]. In breast cancer, a cytoplasmic lncRNA, LINC00478, negatively regulates MYC signaling, thereby inhibiting metastasis [106].

**Figure 1:**
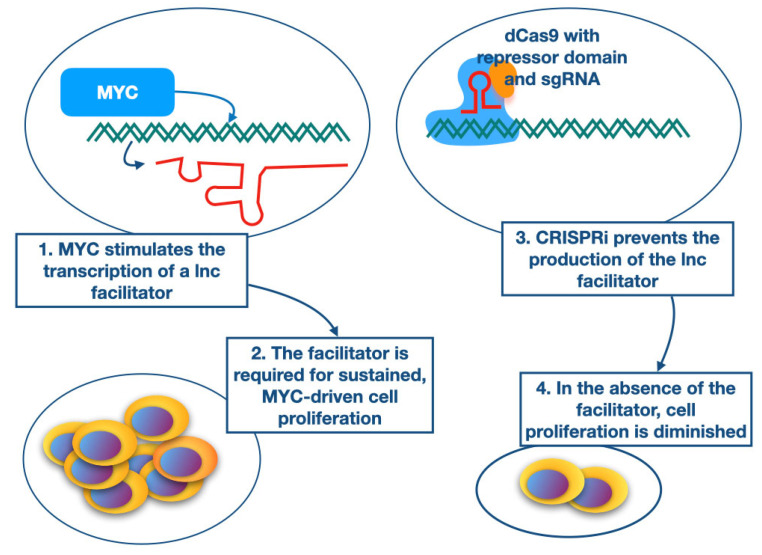
Characteristics of MYC facilitators. The gene encoding the MYC facilitator is a direct target of MYC, and its expression is upregulated by MYC. The lnc transcript of the gene is required for sustained and vigorous MYC-driven cell proliferation. Downregulation of the production of the MYC facilitator by CRISPRi or CRISPR–Cas9 results in diminished cell proliferation.

In this review, we have focused on a specific class of MYC-interacting lncRNAs that we refer to as “MYC facilitators”. We discuss two examples of this category: SNHG17 and LNROP. The common characteristics of these lncRNAs are as follows: (1) They are direct transcriptional targets of MYC, as shown by the binding of MYC to their promoters. (2) Their expression is coordinated with the expression of MYC. (3) They are required for sustained MYC-driven cell proliferation. (4) They are not immediately essential for cell survival [46,58,84]. A summary of these characteristics is presented in Figure 1.

In the human cell lines P496-3 and Ramos, more than 500 MYC facilitators have been identified [82,84]. Although both cell lines are nominally human B cells, their populations of facilitator lncRNAs, though overlapping, are not identical. There are Ramos- and P496-3-specific transcripts. Considering this extreme cell-type specificity of lncRNA expression, the number of MYC facilitators encoded by the human genome could be very large, possibly in the hundreds of thousands.

**Figure 2:**
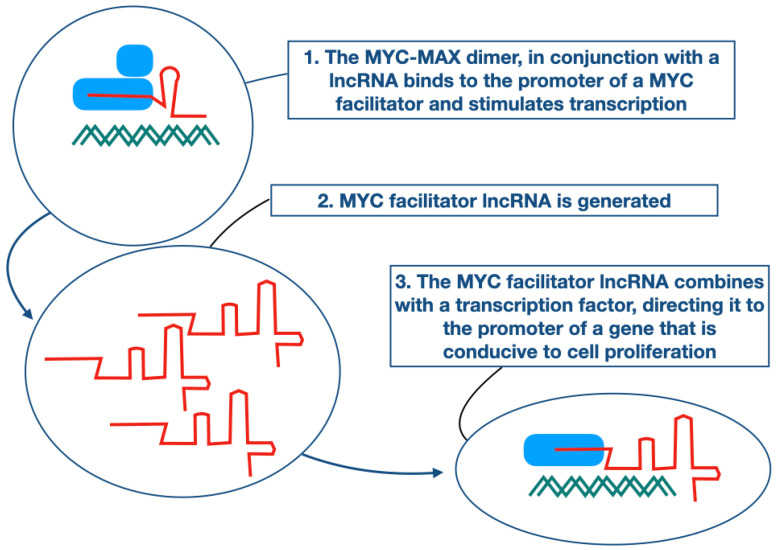
Hypothetical interpretation. MYC is bound to an lncRNA that directs it to the transcriptional start site of a MYC facilitator gene, thus stimulating the transcription of this gene. The MYC facilitator RNA then binds to MYC or another transcription factor and directs it to the transcriptional start site of a growth-promoting gene.

For the observations on MYC facilitators, we offer the following hypothetical explanation: When MYC interacts with the promoter of a MYC facilitator, it is in complex with an lncRNA that provides specificity and accuracy for this interaction by RNA–DNA base pairing. The lncRNA that is generated by this process then contributes to cell proliferation. This contribution is again based on the formation of a complex between protein and RNA. It consists of the newly generated lncRNA and MYC or another transcription factor, and this complex specifically targets cellular genes that promote proliferation. This hypothetical process is summarized in Figure 2. The salient point of this hypothesis is that the regulatory activity of MYC gains precision in promoter targeting through complex formation with a lncRNA.

This proposal could be expanded to include all transcriptional regulation mediated by MYC. For some time, the binding of DNA by MYC has been considered to be determined solely by the sequence of the E-box [107,108]. However, the E-box is a generic binding site that can be accessed by several bHLH transcription factors [109,110]. This two-component interaction cannot account for the precision that is observed in MYC-mediated transcriptional regulation. The idea that MYC acts in concert with RNA is strongly supported by recent experimental data that show transcription factors binding to RNA in vitro and in vivo and that these protein–RNA interactions are essential for function [111]. Both MYC and lncRNAs contain intrinsically disordered regions that could mediate the interactions of MYC with multiple lncRNAs [7,21,39]. Furthermore, the structures of lncRNAs are modular, which would facilitate the combination of an MYC-binding module with a promoter-specifying module and assure the precision of transcriptional regulation [39,112,113]. This proposal is admittedly based on several untested assumptions, but it is accessible via experimental examination. It has the potential to change our views on the transcriptional regulatory activity of MYC.
